# Two Cases of Spontaneous Recurrent Hemarthrosis of the Shoulder with Acromial Erosion Associated with Impingement Syndrome

**DOI:** 10.1155/2019/3042475

**Published:** 2019-02-21

**Authors:** Shoji Fukuta, Katsutoshli Miyatake, Tetsuya Matsuura, Koichi Sairyo

**Affiliations:** ^1^Department of Orthopaedic Surgery, National Hospital Organization Kochi National Hospital, Japan; ^2^Department of Orthopaedic Surgery, Yoshinogawa Medical Center, Japan; ^3^Department of Orthopedics, Tokushima University, Japan

## Abstract

Spontaneous recurrent hemarthrosis of the shoulder is rare. Most previously reported cases were associated with massive rotator cuff tear and degenerative glenohumeral arthritis. We described two cases of recurrent hemarthrosis without osteoarthritis of the shoulder. Both cases had bony erosion of the acromion, which was confirmed arthroscopically as the origin of bleeding. Arthroscopic coagulation, acromioplasty, and drainage were successful and there was no recurrence of hemorrhage.

## 1. Introduction

Spontaneous recurrent hemarthrosis is often seen in the knee joint [1] but it is relatively rare in the shoulder. Synovectomy was commonly performed for recurrent hemarthrosis that had failed to resolve with conservative therapy. Spontaneous hemarthrosis is mostly associated with cuff tear, arthropathy with degenerative change in the glenohumeral joint and attrition of the acromion [2–5]. We encountered two cases of a tense shoulder hemarthrosis associated with unendurable pain and without arthritic changes in the shoulder. Arthroscopic coagulation, acromioplasty, and drainage were successful in both cases.

## 2. Case Reports

### 2.1. Case 1

**Figure 1:**
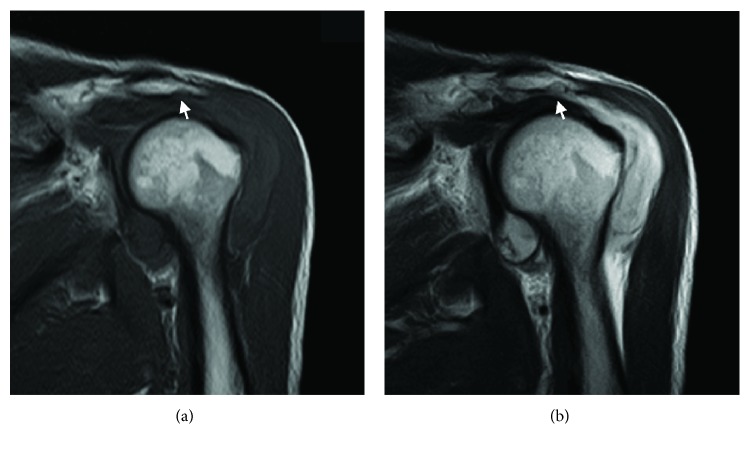
Oblique coronal magnetic resonance images of the left shoulder. (a) T1-weighted images. (b) T2-weighted images. An extensive hematoma was seen in the glenohumeral joint and the subacromial and subdeltoid bursas, which was shown as low signal intensity in T1-weighted images and high signal intensity in T2-weighted images. Bony erosion on the undersurface of the acromion was detected (white arrows).

The patient was a 58-year-old man with 1-month history of pain in the left shoulder with no history of trauma. He had been treated for rotator cuff tendinopathy at a local orthopedic clinic with nonsteroidal analgesics. The pain had worsened 6 hours before his arrival at the emergency department. He had no hypotensive drugs or anticoagulant agents. Physical examination could not be performed due to motion pain and the range of movement of the left shoulder was severely limited in all directions. Radiographs showed irregularity of the greater tuberosity and the undersurface of the acromion but the acromiohumeral distance was normal. MRI demonstrated large hematomas in both the glenohumeral joint and the subacromial space and bony erosion of the lateral acromion (Figure 1). Pain was relieved by aspiration of approximately 45 mL of blood. Six hours later, he revisited the emergency department because of recurrence of unendurable pain.

**Figure 2:**
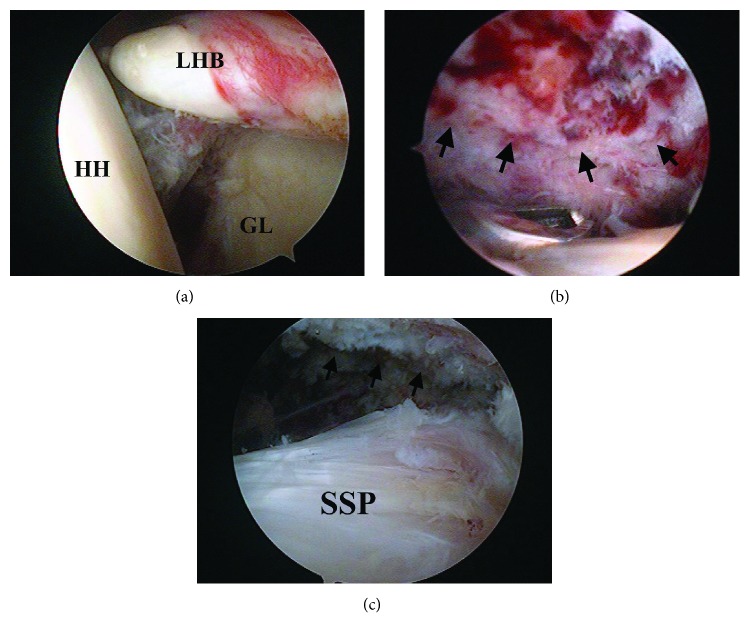
Arthroscopic views. (a) In the glenohumeral joint, the long head of the biceps was torn and the intra-articular portion had disappeared (LHB: long head of the biceps; GL: glenoid; HH: humeral head). (b) Bleeding was noted from the subacromial erosion (arrows). (c) Acromioplasty was performed (arrows) following coagulation of the bleeding points. The rotator cuff was intact (SSP: supraspinatus).

Arthroscopy was performed on the same day to confirm the diagnosis and control bleeding. There were no abnormal findings in the glenohumeral joint except for a chronic tear of the biceps tendon (Figure 2(a)). There was a defect in the rotator interval but the supraspinatus tendon was intact. Proliferative synovitis was not observed in the subacromial bursa and no tumorous lesions such as pigmented villonodular synovitis (PVNS) were seen. A crater-like erosion was observed on the undersurface of the acromion, at which bleeding was seen (Figure 2(b)). Bleeding points were coagulated with a radiofrequency system and anterior acromioplasty was performed with a motorized shaver arthroscopically (Figure 2(c)). Abnormal bleeding was not noted after acromioplasty. A drain was placed in the subacromial space through the anterolateral portal before closure and it was removed on the third day after surgery. At the 48-month follow-up, there had been no recurrence of hemarthrosis and the patient was completely pain-free with full range of the shoulder motion. The UCLA score was improved from 3 points preoperatively to 35 points at the final follow-up.

### 2.2. Case 2

**Figure 3:**
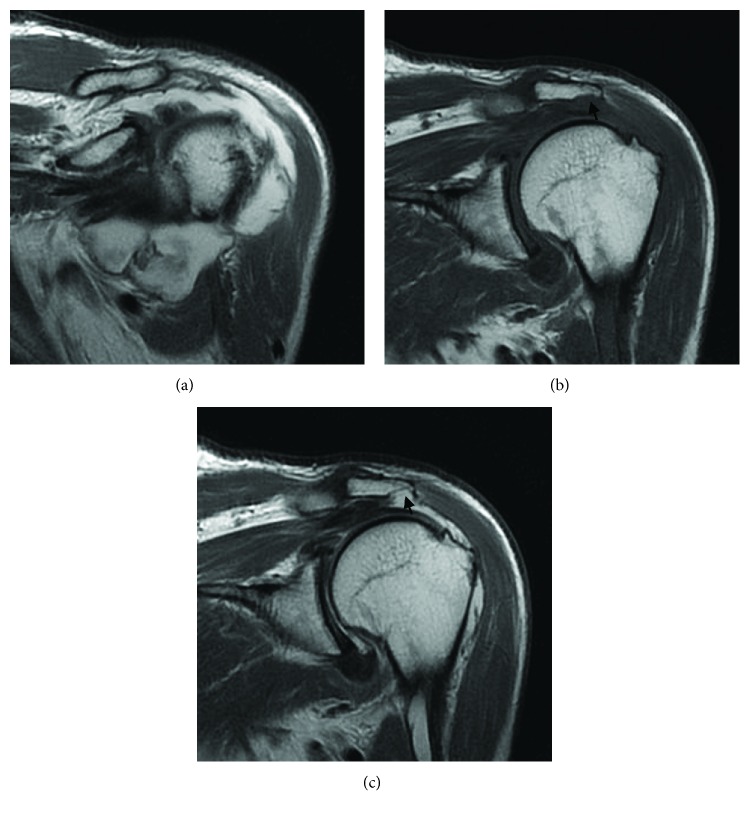
Oblique coronal magnetic resonance images of the left shoulder. A hematoma was seen in the anterior portion of the subacromial bursa in T2-weighted images (a). Subacromial bony erosion was detected in T1- (b) and T2-weighted images (c) (arrows). Bursal-side partial tear was also seen.

A 67-year-old man complained of left shoulder pain for 2 weeks without any history of trauma. Bloody effusions were aspirated 5 times at a local orthopedic clinic during a 2-week period before the patient was referred to us. He had no previous history related to hemorrhagic factors. On examination, he had slightly limited range of motion, with active forward flexion to 160°, abduction to 120°, external rotation to 30°, and internal rotation to the fifth lumbar spine with the arm at the side. Passive range of motion was the same as active range of motion. Neer's impingement test and Hawkins test were positive. The greater tuberosity was seen irregular on radiography. MRI demonstrated a hematoma in the anterior portion of the subdeltoid bursa and bony erosion of the lateral acromion, similar to case 1 (Figure 3).

**Figure 4:**
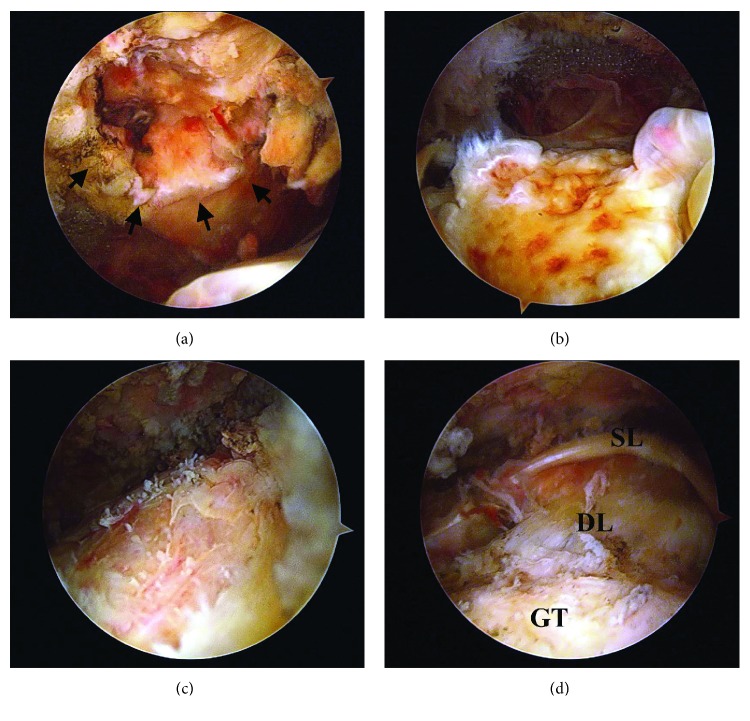
Arthroscopic views. (a) Crater formation on the undersurface of the acromion and bleeding was noted from the subacromial erosion (arrows). (b) Irregularity of the greater tuberosity was observed. (c) Osteophytes of the greater tuberosity were removed by a shaver following coagulation of the bleeding points. (d) Bursal-side partial-thickness tear was not repaired (GT: greater tuberosity; DL: deep layer of the cuff; SL: superficial layer of the cuff).

Arthroscopy was performed to determine the cause of the hemorrhage. A hematoma was not observed in the glenohumeral joint. The intra-articular portion of the long head of the biceps tendon had completely disappeared. A bursal-side partial tear of the rotator cuff was noted. The size of the tear was 25 mm in the anteroposterior direction. Crater formation on the undersurface of the acromion and irregularity of the greater tuberosity were observed, which seemed to be kissing lesions (Figures 4(a) and 4(b)). Active bleeding was seen at the exposed bone marrow of the acromion when the pressure of irrigation was lowered. The undersurface of the acromion was coagulated and the greater tuberosity was abraded to avoid impingement (Figure 4(c)). Acromioplasty was not performed in this case. The bursal-side partial tear was not repaired at that time (Figure 4(d)).

One month later, the patient underwent arthroscopic rotator cuff repair. The patient regained full range of motion 3 months after rotator cuff repair and returned to work as a taxi driver. At the final follow-up, 14 months after the first arthroscopy, he had no functional deficit. The UCLA score was improved from 14 points preoperatively to 35 points at the final follow-up. Hemarthrosis did not recur in the 14 months postoperatively.

## 3. Discussion

Spontaneous hemarthrosis of the shoulder is rare. Hemophilic arthropathy is a well-known pathology that causes hemarthrosis of the shoulder [6]. Recently, hemarthrosis induced by anticoagulant medication has been reported [7]. Both our patients were not hemophilic and had not taken any anticoagulants. PVNS rarely involves the shoulder joint but should be excluded as a cause of hemarthrosis. In both cases, no tumorous lesion such as PVNS was observed. The synovium in the glenohumeral joint and subacromial bursa seemed normal with no diffuse proliferative synovitis, which was often arthroscopically observed in hemarthrosis of the knee [1]. Reactive synovitis mimicking PVNS resulted from chronic intra-articular bleeding [5]. The cases presented here were in the acute phase and the interval from the onset to arthroscopy was short. Therefore, the synovium was not inflamed.

The association between rotator cuff tear and hemarthrosis has been described by several investigators. Banna and Kendall first described three spontaneous hemarthroses in 1964 and proposed hemorrhagic tendinitis as a cause of hemarthrosis. Ishikawa et al. [2] also reported three cases of persistent hemarthrosis with massive rotator cuff tear. Two of the three cases were spontaneous and the other was secondary to a fracture of the anatomical neck of the humerus. They confirmed that the site of bleeding was the synovium and postulated that biological reactions following phagocytosis, as well as anatomical and mechanical derangements, might have contributed to hemorrhage based on the histological findings of the synovium harvested during the surgery.

Sano and Nakajo [4] reported a case of recurrent hemarthrosis that was treated arthroscopically by coagulating the bleeding followed by minimal acromioplasty. They confirmed that the bleeding point was from the center of the chondral defect on the humeral head just medial to the greater tuberosity. They arthroscopically demonstrated that the chondral defect impinged upon the medial edge of the acromion at 50° abduction and concluded that impingement due to osteophyte formation and instability might contribute to attrition of the humeral head. The cases reported previously were associated with massive rotator cuff tear, superior migration of the humeral head, and subsequent instability of the shoulder, which matched the criteria of cuff tear arthropathy proposed by Neer et al. [8]. Spontaneous hemarthrosis associated with cuff tear arthropathy is characterized by rapid onset of severe pain, limited shoulder movement, and upward migration of the humeral head coupled with attrition of the acromion. Differences between our cases and previously described cases exist. Both our patients had no evidence of degeneration in the glenohumeral joint. The rotator cuff was intact or torn partially so that the acromioclavicular distance was maintained. The common findings of the two cases were irregularity of the greater tuberosity and acromial erosion that indicates subacromial impingement. The cause and mechanism of the impingent were not clear in both cases. In case 2, irregular surface of the greater tuberosity directly impinged to the acromion, which formed a crater on its undersurface. We therefore considered that the flattening of the greater tuberosity would be enough to prevent the recurrence. In case 1, acromioplasty was performed additionally because he had intact supraspinatus tendon and lacked bony impingement. Bleeding from the undersurface of the acromion was confirmed arthroscopically, which might be the cause of hemarthrosis. Soft tissue was attached to the undersurface of the acromion, but the origin of the bleeding seemed to be a bone marrow because soft tissue did not contact with the bursal synovial tissue.

Hemarthrosis with cuff tear arthropathy often requires reverse total shoulder arthroplasty. However, arthroscopic treatment is recommended in nonarthritic patients when the recurrent hemarthrosis does not respond to several aspirations. Arthroscopy is especially useful in patients without a complete rotator cuff tear. It allows the examination of both the glenohumeral joint and subacromial bursa without sacrificing the rotator cuff. Only one previous report had managed shoulder hemarthrosis arthroscopically [4]. In our cases, we performed coagulation and drainage to relieve pain by decreasing the pressure in the subacromial bursa. Acromioplasty was added to improve impingement and prevent the recurrence of hemarthrosis. Arthroscopy was also useful to determine the origin of bleeding and differential diagnosis.

## References

[1] Sasho T., Ogino S., Tsuruoka H. (2008). Spontaneous recurrent hemarthrosis of the knee in the elderly: arthroscopic treatment and etiology. *Arthroscopy*.

[2] Banna A., Kendall P. H. (1964). Spontaneous haemarthrosis of the shoulder joint. *Rheumatology*.

[3] Ishikawa K., Ohira T., Morisawa K. (1988). Persistent hemarthrosis of the shoulder joint with a rotator-cuff tear in the elderly. *Archives of Orthopaedic and Traumatic Surgery*.

[4] Sano H., Nakajo S. (2004). Repeated hemarthrosis with massive rotator cuff tear. *Arthroscopy*.

[5] France M. O., Gupta S. K. (1991). Nonhemophilic hemosiderotic synovitis of the shoulder. A case report. *Clinical Orthopaedics and Related Research*.

[6] Chen Y. C., Chen L. C., Cheng S. N., Pan R. Y., Chang S. T., Li T. Y. (2013). Hemophilic arthropathy of shoulder joints: clinical, radiographic, and ultrasonographic characteristics of seventy patients. *The Journal of Bone and Joint Surgery*.

[7] Davis C. B., Nowak R. M. (2014). Anticoagulant-induced hemarthrosis presenting as anterior shoulder dislocation. *The American Journal of Emergency Medicine*.

[8] Neer C. S., Craig E. V., Fukuda H. (1983). Cuff-tear arthropathy. *The Journal of Bone & Joint Surgery*.

